# Identification of a sub-population of B cells that proliferates after infection with epstein-barr virus

**DOI:** 10.1186/1743-422X-8-84

**Published:** 2011-02-25

**Authors:** Cynthia Megyola, Jianjiang Ye, Sumita Bhaduri-McIntosh

**Affiliations:** 1 Department of Pediatrics, Yale University School of Medicine, New Haven, CT 06520, USA; 2 Department of Molecular Biophysics and Biochemistry, Yale University School of Medicine, New Haven, CT 06520, USA; 3 Current Address: Department of Genetics, Yale University School of Medicine, New Haven, CT 06520, USA; 4 Current Address: Fei Tian Academy of the Arts, Cuddebackville, NY 12729, USA; 5 Current Address: Departments of Pediatrics, Molecular Genetics and Microbiology, State University of New York at Stony Brook, Stony Brook, NY 11794, USA

## Abstract

**Background:**

Epstein-Barr virus (EBV)-driven B cell proliferation is critical to its subsequent persistence in the host and is a key event in the development of EBV-associated B cell diseases. Thus, inquiry into early cellular events that precede EBV-driven proliferation of B cells is essential for understanding the processes that can lead to EBV-associated B cell diseases.

**Methods:**

Infection with high titers of EBV of mixed, primary B cells in different stages of differentiation occurs during primary EBV infection and in the setting of T cell-immunocompromise that predisposes to development of EBV-lymphoproliferative diseases. Using an *ex vivo *system that recapitulates these conditions of infection, we correlated expression of selected B cell-surface markers and intracellular cytokines with expression of EBV latency genes and cell proliferation.

**Results:**

We identified CD23, CD58, and IL6, as molecules expressed at early times after EBV-infection. EBV differentially infected B cells into two distinct sub-populations of latently infected CD23^+ ^cells: one fraction, marked as CD23^hi^CD58^+^IL6^- ^by day 3, subsequently proliferated; another fraction, marked as CD23^lo^CD58^+^, expressed IL6, a B cell growth factor, but failed to proliferate. High levels of LMP1, a critical viral oncoprotein, were expressed in individual CD23^hi^CD58^+ ^and CD23^lo^CD58^+ ^cells, demonstrating that reduced levels of LMP1 did not explain the lack of proliferation of CD23^lo^CD58^+ ^cells. Differentiation stage of B cells did not appear to govern this dichotomy in outcome either. Memory or naïve B cells did not exclusively give rise to either CD23^hi ^or IL6-expressing cells; rather memory B cells gave rise to both sub-populations of cells.

**Conclusions:**

B cells are differentially susceptible to EBV-mediated proliferation despite expression of viral gene products known to be critical for continuous B cell growth. Cellular events, in addition to viral gene expression, likely play a critical role in determining the outcome of EBV infection. By indentifying cells predicted to undergo EBV-mediated proliferation, our study provides new avenues of investigation into EBV pathogenesis.

## Background

Infection of B cells with Epstein-Barr virus (EBV) leads to proliferation and subsequent immortalization, resulting in establishment of lymphoblastoid cell lines *in vitro *(LCL). LCL-like cells are observed during primary EBV infection [1], in tonsils of healthy individuals [2,3], and are characteristic of EBV-associated lymphomas and lymphoproliferative diseases in immunocompromised hosts [4]. EBV-driven B cell proliferation is essential for development of such tumors *in vivo *and for outgrowth into LCL *ex vivo*. A large body of evidence has established that EBV proteins EBNA2 and LMP1 [5-10] are critical viral oncoproteins that are required for growth transformation of B cells. Regarding cellular events that follow EBV infection, with some exceptions [6,11], most studies have focused on late events such as outgrowth of LCL, 3 to 8 weeks after infection with EBV [12,13]. While CD23, the low affinity receptor for IgE, was found to be expressed early on cells undergoing immortalization [6,11], little is known about the exact relationship between CD23 expression at early times, expression of viral latency genes, and subsequent proliferation or immortalization [14,15]. To better understand EBV pathogenesis, it is important to dissect early host cell processes that precede EBV-driven B cell proliferation.

According to a well-supported model [16], primary infection with EBV in healthy individuals and the early stages of development of B cell-EBV lymphoproliferative diseases/lymphomas in immunocompromised hosts are characterized by infection of polyclonal B cells in different stages of differentiation by high titers of EBV in the absence of EBV-specific protective immune responses. Using an *ex vivo *system that emulates these conditions, we sought the identity of B cells that underwent proliferation after exposure of total peripheral B cells to high titers of EBV in the absence of EBV-specific immune responses. B cell surface markers, intracellular cytokines, and expression of EBV genes were interrogated simultaneously and correlated with cell proliferation to identify a specific sub-population of B cells susceptible to EBV-driven proliferation.

## Results

### Exposure to EBV results in differential levels of expression of CD23

**Figure 1:**
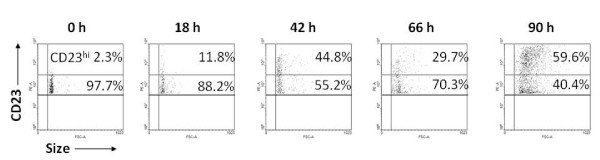
**Exposure to EBV results in differential levels of expression of CD23 on B cells**. Expression of CD23 was determined using PE-anti-CD23 antibody at 0 h, 18 h, 42 h, 66 h, and 90 h after exposure of B cells to EBV. Percent CD23^+ ^cells expressing low levels or high levels of CD23 (CD23^hi^) are shown.

B cells undergoing EBV-driven immortalization express high levels of CD23 [6,7]. We examined the kinetics of expression of CD23 on CD23^+ ^cells at early times following exposure of total primary B cells to EBV. Figure 1 shows that following exposure to EBV, the fraction of CD23^+ ^cells that expressed high levels of CD23 (CD23^hi^) increased. There was a rapid shift in the pattern of CD23 expression from 2.3% CD23^hi ^cells at time 0 to 59.6% CD23^hi ^cells at 90 h resulting in a 25.9-fold increase at 90 h relative to time 0. Therefore, exposure to EBV resulted in sub-populations of B cells with differential levels of expression of CD23. No alterations in expression of CD23 from baseline were observed when un-infected cells were cultured (data not shown).

### CD58, CD23, and IL6, the earliest expressed molecules, mark the emergence of distinct sub-populations of B cells after exposure to EBV

To distinguish distinct sub-populations within CD23^+ ^B cells early after exposure to EBV, we correlated expression of CD23 with expression of other B cell markers including CD58, IL6, CD57, CD86, HLA Class II, PD1, and IL10. Table 1 shows that there was a rapid increase in the fraction of CD23^+ ^cells that expressed CD58 in the first 18 h relative to time 0 (9.7-fold in subject 1 and 21.5-fold in subject 2). While nearly a third of CD23^- ^cells expressed CD58 at time 0, there was less than a two-fold increase over the duration of the experiments. Thus among CD23^+ ^cells, there was a rapid increase in expression of CD58.

**Table 1:** Percent CD23^+^/CD23^- ^cells expressing surface molecules or cytokines after exposure of B cells to EBV

Subject 1										
	**0 hr**	**18 hr**	**42 hr**	**66 hr**	**90 hr**	**0 hr**	**18 hr**	**42 hr**	**66 hr**	**90 hr**

	**CD23^+^**					**CD23^-^**				

CD58	1.5	**14.5**	59.3	50.9	81.2	30.5	45.6	64.9	57.8	59.3

IL6	0.5	2.5	6.8	**13.5**	44	0.5	0.2	0.2	1.7	1.3

CD57	1	5.4	15.8	27.3	43.2	9.7	8.2	6.5	10.3	8.3

CD86	17.6	23.2	21.2	35.3	38.5	24.5	23.6	36.7	60.4	63.2

MHC CII	99.9	99.6	99.5	99.7	99.9	72.2	75.1	71.8	74.8	69

PD1	14.2	10.8	8.5	9.1	6	5.6	5.6	4.8	6.1	8.6

IL10	0.2	0.1	0.1	0.07	0.09	0.1	0.08	0.09	0.06	0.1

**Subject 2**										

CD58	1	**21.5**	22.1	38	45.1	39.9	47.6	57.1	54.3	57.6

IL6	0.6	1.6	2.8	**14.5**	20.7	0.7	1.6	0.9	0.9	0.4

CD57	1.4	3.5	5.3	8.9	17.7	17.6	18.2	14.9	13.7	12.5

CD86	16.8	17.4	15.9	36.2	48.8	34.3	36.5	51.4	54.5	57.5

MHC CII	99.4	99.1	99.2	99.3	99.7	59.2	57.2	61.7	63.4	63.8

PD1	1.8	1.7	1.1	3.9	5.8	2.3	2.1	1	1.7	2.8

IL10	0.1	0.1	0.1	0.04	0.2	0.03	0.04	0.04	0.05	0.08

Intracellular expression of IL6 increased in CD23^+ ^cells by 42 to 66 h (15.8-fold at 42 h in subject 1 and 24.1-fold at 66 h in subject 2) as compared to time 0. Very few CD23^- ^cells expressed IL6. CD57 expression increased substantially on CD23^+ ^cells (15.8-fold) at 42 h in only subject 1. No substantial change in expression on CD23^+ ^cells was observed for the other molecules. Although levels of expression of CD58 (LFA3), an adhesion molecule, and the cytokine IL6 altered most rapidly following exposure to EBV, expression of CD58 on CD23^+ ^cells was the earlier marker of infection with EBV.

**Figure 2:**
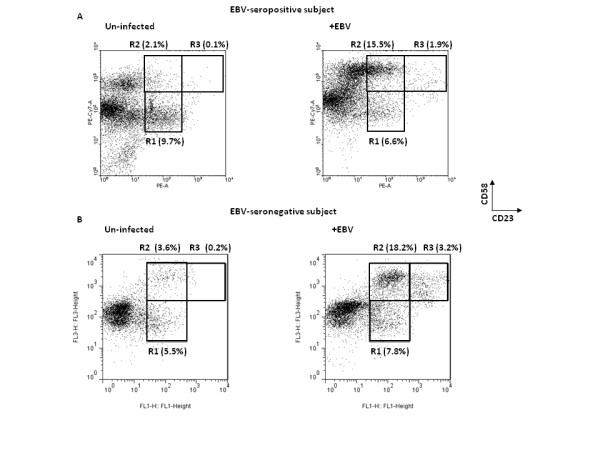
**Emergence of distinct sub-populations of B cells expressing CD23 and CD58 following exposure to EBV**. B cells from a representative healthy EBV-seropositive (A) and a representative healthy EBV-seronegative (B) subject were un-infected (left panels) or exposed to EBV (right panels) and placed in culture. Cells were harvested on day 4 and examined for surface expression of CD23 and CD58. Percent cells in regions R1 (CD23^lo^CD58^-^), R2 (CD23^lo^CD58^+^), and R3 (CD23^hi^CD58^+^) are shown.

Next, we examined whether there was a correlation between the level of expression of CD23 and CD58. Figure 2 shows B cells from a representative healthy EBV-seropositive subject (A) and a representative healthy EBV-seronegative subject (B) that were un-treated or exposed to EBV for four days. When compared with un-infected cells, two distinct sub-populations of CD23^+ ^cells emerged after EBV infection. Sub-population R2 was characterized by expression of CD58 and low levels of expression of CD23 (CD23^lo^CD58^+^) while sub-population R3 expressed CD58 and high levels of CD23 (CD23^hi^CD58^+^). Thus, two distinct sub-populations of B cells could be identified based on presence of CD58 and either high or low level of expression of CD23 early after exposure to EBV of cells from both EBV-seropositive and -seronegative individuals.

### Expression of CD58 but not IL6 correlates with proliferation of EBV-exposed cells

**Figure 3:**
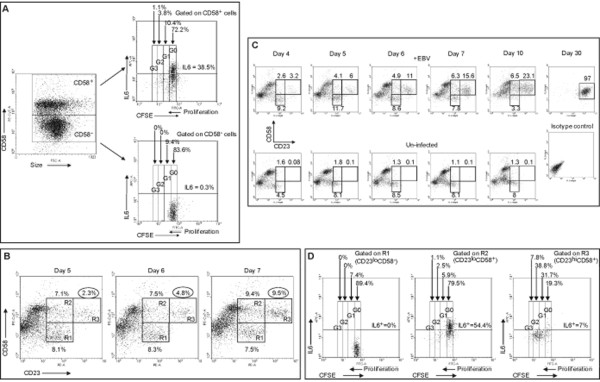
**CD23^hi^CD58^+ ^cells proliferate and constitute the bulk of the population by day 30**. A. Carboxyfluorescein diacetate, succinimidyl ester (CFSE)-labeled B cells were exposed to EBV and harvested on day 5. CD58^+ ^cells or CD58^- ^cells were examined for proliferation and intracellular expression of IL6 (using APC-anti-IL6 antibody). Percentages represent fraction of gated cells (CD58^+ ^or CD58^-^) producing IL6. G0-3 represents non-proliferated cells (G0) and three generations of progeny (G1-3). Percentages above G0-3 indicate the fraction of CD58^+ ^or CD58^- ^cells in each generation. B. EBV-exposed B cells were harvested on days 5, 6 and 7, and examined for expression of CD23 (PE) and CD58 (PE-Cy7). Percentages represent fractions of cells in regions R1 (CD23^lo^CD58^-^), R2 (CD23^lo^CD58^+^), and R3 (CD23^hi^CD58^+^). C. In a separate experiment, EBV-exposed cells (top panels) were harvested on days 4, 5, 6, 7, 10, and 30 and un-infected cells (bottom five panels) were harvested on days 4, 5, 6, 7, and 10 and examined for expression of CD23 and CD58. Percentages of cells in regions R1, R2, and R3 are shown. EBV-exposed cells harvested on day 30 and stained with PE and PE-Cy7 isotype control antibodies are also shown. D. CFSE-labeled B cells were exposed to EBV, harvested on day 5, and examined for expression of CD23 (PE) and CD58 (PE-Cy7). Cells in region R1, R2, and R3 were examined for proliferation and expression of IL6 (APC) by flow cytometry. Percentages represent fraction of cells in regions R1, R2, or R3 that produced IL6. Percentages above G0-3 indicate non-proliferating cells (G0) and proliferating cells (G1-3).

Since CD58 and IL6 were expressed early on CD23^+ ^cells, we asked if any of these markers correlated with proliferation of cells. To examine proliferation, we labeled B cells with CFSE prior to exposure to EBV. CFSE is redistributed equally among daughter cells resulting in approximate halving of fluorescence intensity with each round of proliferation. A representative experiment shows that five days after exposure to EBV, 15.3% (G1+G2+G3) of CD58^+ ^cells had proliferated (Figure 3A) and approximately 5% of cells had undergone more than one round of proliferation. In comparison, none of the CD58^- ^cells had undergone more than one round of proliferation. Among CD58^+ ^cells, 38.5% expressed IL6 while only 0.3% of CD58^- ^cells expressed IL6. Thus, although CD58 expression was associated with expression of IL6, proliferating cells expressed CD58 but not IL6. Results of Figure 3A show that there were at least three sub-groups of CD58^+ ^cells: one group that proliferated but did not express IL6, another that expressed IL6 but did not proliferate, and a third that did neither.

### The expression pattern CD23^hi^CD58^+^IL6^- ^marks cells that proliferate

To determine how expression of CD58 correlated with proliferation, we temporally followed the evolution of the two CD23^+^CD58^+ ^sub-populations and the CD23^+^CD58^- ^sub-population after exposure to EBV. Figure 3B shows that there was an approximate doubling in the fraction of cells in R3 (CD23^hi^CD58^+ ^cells) from 2.3% to 4.8% between day 5 and day 6 and from 4.8% to 9.5% between day 6 and day 7. In contrast, the two other sub-populations R1 (CD23^lo^CD58^- ^cells) and R2 (CD23^lo^CD58^+ ^cells) did not show a similar doubling. Tracking the three sub-populations from day 4 to day 30 revealed a progressive increase in the fraction of CD23^hi^CD58^+ ^cells resulting in the exclusive presence of CD23^hi^CD58^+ ^cells by day 30 (Figure 3C, upper panels). Un-infected cells showed no outgrowth of LCL (Figure 3C, lower panels) and were dead after 10 days by Trypan blue exclusion (data not shown). These findings suggested that CD23^hi^CD58^+ ^cells were likely to be proliferating. Figure 3C also shows that neither CD23^lo^CD58^+ ^cells nor CD23^hi^CD58^+ ^cells emerged when B cells from EBV-seropositive individuals were placed in culture in the absence of exogenously added EBV.

Proliferation of cells in each sub-population was examined by exposure of CFSE-labeled B cells to EBV for five days (Figure 3D). Nearly 80% of CD23^hi^CD58^+ ^cells (R3) had proliferated with 31.7% cells in G1, 38.8% of cells in G2, and 7.8% of cells in G3. In contrast, the vast majority of CD23^lo^CD58^- ^cells (89.4% in R1) and CD23^lo^CD58^+ ^cells (79.5% in R2) had not proliferated. The earliest time at which proliferation was observed was four days after exposure to EBV (data not shown). Simultaneous examination for expression of IL6 revealed that 54.4% of CD23^lo^CD58^+ ^cells expressed IL6. Minimal to no IL6 expression was observed in CD23^hi^CD58^+ ^cells (7%) and CD23^lo^CD58^- ^cells (0%). Thus, expression of IL6 and proliferation were mutually exclusive (Figure3A and 3D). The expression pattern CD23^hi^CD58^+^IL6^- ^was characteristic of cells that underwent proliferation.

### CD23^hi^CD58^+ ^cells do not proliferate in the absence of EBV-exposed non-proliferating sub-populations of cells

**Figure 4:**
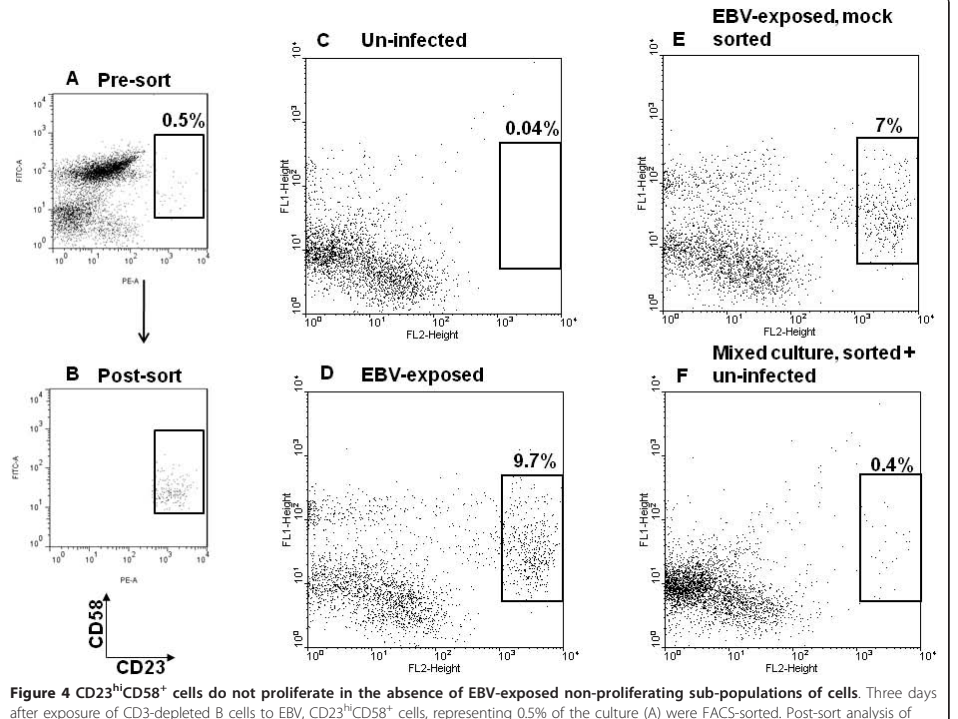
**CD23^hi^CD58^+ ^cells do not proliferate in the absence of EBV-exposed non-proliferating sub-populations of cells**. Three days after exposure of CD3-depleted B cells to EBV, CD23^hi^CD58^+ ^cells, representing 0.5% of the culture (A) were FACS-sorted. Post-sort analysis of CD23^hi^CD58^+ ^cells is shown in B. Sorted CD23^hi^CD58^+ ^cells were mixed with un-infected autologous primary B cells (as feeder cells) and re-introduced into culture at 0.5% of the total culture. Mock-sorted cells were also re-introduced into culture as control. Cells were harvested four days later and stained for CD23 (PE) and CD58 (FITC). Un-infected cells (C), EBV-exposed cells (D), mock-sorted but EBV-exposed cells (E), and sorted-CD23^hi^CD58^+ ^cells mixed with un-infected B cells (F) after a total of 7 days in culture are shown. Percentages represent fraction of CD23^hi^CD58^+ ^cells out of total.

We examined whether CD23^hi^CD58^+ ^cells were able to proliferate in the absence of EBV-exposed non-proliferating B cells. Three days after exposure to EBV, FACS-sorted CD23^hi^CD58^+ ^cells, representing 0.5% of the culture, were re-introduced into culture after mixing with un-infected autologous primary B cells as feeder cells to maintain CD23^hi^CD58^+ ^cells at 0.5% of the culture. Three day old pre-sort culture and post-sort CD23^hi^CD58^+ ^cells are shown in Figure 4A and B, respectively. Un-infected, EBV-exposed, EBV-exposed but mock-sorted, and mixed culture of sorted-CD23^hi^CD58^+ ^cells plus un-infected cells were harvested on day 7. Un-infected B cells gave rise to only 0.04% CD23^hi^CD58^+ ^cells (C). Un-disturbed EBV-exposed B cells gave rise to 9.7% (D) and mock-sorted EBV-exposed B cells gave rise to 7% (E) CD23^hi^CD58^+ ^cells demonstrating that manipulation of cells during staining and sorting did not substantially hinder proliferation of CD23^hi^CD58^+ ^cells. In contrast, the percentage of CD23^hi^CD58^+ ^cells four days after mixing sorted-CD23^hi^CD58^+ ^cells with un-infected cells remained unchanged (F) as compared to the fraction of CD23^hi^CD58^+ ^cells in the pre-sorted population (A) suggesting dependence of CD23^hi^CD58^+ ^cells on the non-proliferating EBV-exposed sub-populations.

### CD23^hi ^cells and IL6^+ ^cells can both be derived from memory B cells

**Figure 5:**
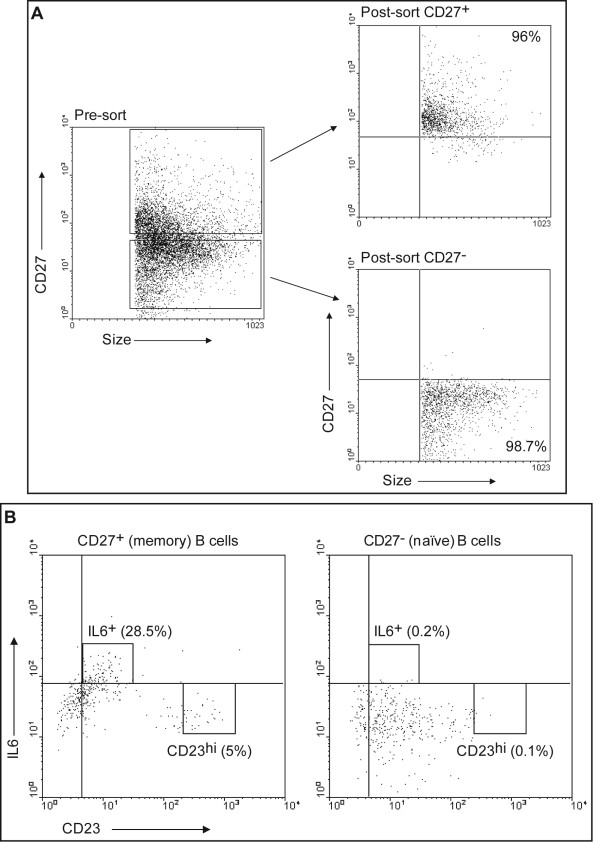
**CD23^hi ^cells and IL6^+ ^cells are derived from CD27^+ ^memory B cells**. A. PBMC were FACS-sorted into CD27^+ ^(memory) and CD27^- ^(naïve) B cells. Sorting strategy and purity of cells after sorting are shown. B. Sorted CD27^+ ^and CD27^- ^B cells were exposed to EBV. Cells were harvested on day 5 and examined for expression of CD23 (FITC) and intracellular IL6 (APC) by flow cytometry. Percentages represent IL6^+ ^or CD23^hi ^cells of CD27^+ ^or CD27^- ^B cells.

We asked whether susceptibility to proliferation or IL6 expression was pre-determined by the differentiation state of B cells before infection. Peripheral B cells were FACS-sorted into CD27^+ ^memory and CD27^- ^naïve B cells. CD27 is considered a general marker for peripheral memory B cells [17,18]. Sorting strategy and purity of sorted populations of a representative experiment are shown in Figure 5A. Simultaneous staining of B cells with CD27 or matched isotype control antibody allowed detection of CD27^+ ^cells prior to sort (data not shown). Sorted cells were examined on day 5 after exposure to EBV for expression of CD23 and IL6 (Figure 5B). Since CD23^hi^CD58^+ ^cells underwent proliferation (Figure 3D) and nearly all CD23^hi ^cells also expressed CD58 after exposure to EBV (Figure 2), 5% of memory B cells (CD23^hi^) were predicted to proliferate after exposure to EBV (Figure 5B). In comparison, only 0.1% of naïve B cells were CD23^hi^. Nearly 30% of memory B cells but only 0.2% of naïve B cells expressed IL6. Among memory B cells, expression of IL6 and expression of high levels of CD23 were mutually exclusive. Both sub-populations of B cells, whether susceptible to proliferation or expression of IL6, were derived from memory B cells. Thus, memory or naïve B cells did not exclusively give rise to either proliferating or IL6-expressing cells.

### Correlations between proliferation, expression of IL6, and EBV latency gene expression

**Figure 6:**
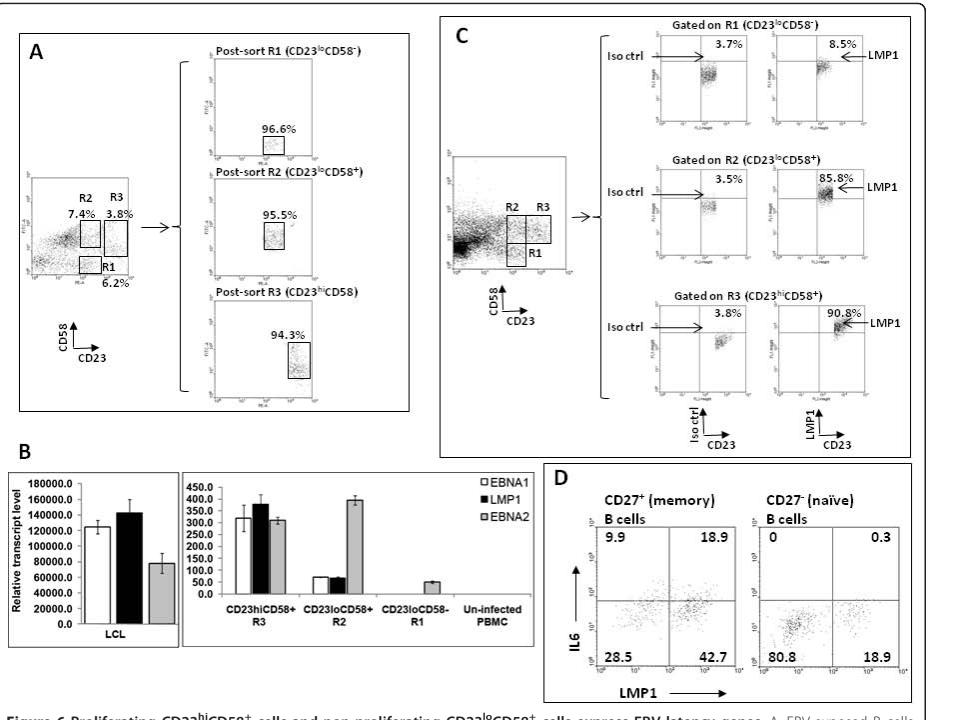
**Proliferating CD23^hi^CD58^+ ^cells and non-proliferating CD23^lo^CD58^+ ^cells express EBV latency genes**. A. EBV-exposed B cells were FACS-sorted on day 4 into regions R1 (CD23^lo^CD58^-^), R2 (CD23^lo^CD58^+^), and R3 (CD23^hi^CD58^+^). Sorting strategy and purity of each population are shown. Regions were drawn with spaces in between to prevent contamination between regions. B. The relative transcript levels of latency genes EBNA1, LMP1, and EBNA2 in each of the three sorted sub-populations were determined by real-time reverse transcription-PCR (qRT-PCR) with gene-specific primers. RNA from already immortalized cells (LCL) from the same subject was used as positive control while RNA from un-infected PBMC from the same subject was used as negative control. C. EBV-exposed B cells were harvested on day 3 followed by staining for CD23 (PE), CD58 (PE-Cy7), and intracellular LMP1 (FITC) and flow cytometry. Expression of LMP1 in cells gated on R1, R2, and R3 is shown. Percentages represent fractions of gated cells expressing LMP1 or detected by the corresponding isotype control antibody. D. Sorted CD27^+ ^memory and CD27^- ^naïve B cells as in the experiment shown in Fig. 5B were exposed to EBV, harvested on day 5, and examined for expression of intracellular IL6 (APC) and LMP1 (PE). Numbers represent percentages of CD27^+ ^memory or CD27^- ^naïve B cells.

To determine which sub-populations of CD23^+ ^cells expressed viral latency genes, CD23^lo^CD58^- ^(R1), CD23^lo^CD58^+ ^(R2), and CD23^hi^CD58^+ ^(R3) cells were FACS-sorted four days after exposure of B cells to EBV. A representative experiment showing sorting strategy and purity of sorted sub-populations is shown in Figure 6A. Both CD23^hi^CD58^+ ^and CD23^lo^CD58^+ ^sub-populations expressed EBNA1, LMP1, and EBNA2 transcripts, a pattern consistent with viral type 3 latency which is characteristic of EBV-immortalized B cells (Figure 6B). In comparison, CD23^lo^CD58^- ^cells expressed only EBNA2 mRNA. Since expression of mRNA often does not correlate well with expression of protein and because qRT-PCR is not informative about the fraction of cells expressing a gene of interest or the level of expression at the single cell level, we examined EBV-exposed cells for expression of LMP1 protein at the earliest possible time, on day 3, by flow cytometry. Figure 6C shows that 87% of CD23^hi^CD58^+ ^cells and 82.3% of CD23^lo^CD58^+ ^cells expressed similar high levels of LMP1 when compared with isotype control antibody-staining of corresponding sub-populations. In contrast, only 4.8% of CD23^lo^CD58^- ^cells expressed low levels of LMP1. Sorted cells also demonstrated a similar pattern of expression of LMP1 in CD23^hi^CD58^+ ^and CD23^lo^CD58^+ ^cells by immunofluorescence (data not shown). Thus, both distinct sub-populations of cells namely CD23^hi^CD58^+ ^IL6^- ^and CD23^lo^CD58^+ ^IL6^+ ^expressed viral latency genes including LMP1, a critical oncoprotein necessary for growth transformation in LCL[19]; yet only one of these populations proliferated. This data also demonstrated that lack of proliferation of CD23^lo^CD58^+ ^cells was not due to lack of transition from an early EBNA2-expression stage to subsequent LMP1-expression in this sub-population. Both proliferating and IL6-expressing sub-populations also expressed high levels of EBNA2 transcript. Characteristics of the two sub-populations CD23^hi^CD58^+^IL6^- ^and CD23^lo^CD58^+^IL6^+ ^are shown in Table 2.

**Table 2:** Characteristics of the two sub-populations of CD23^+ ^cells that emerge after exposure to EBV

	Proliferating	Non-proliferating
CD23	hi	lo

CD58	+	+

IL6	-	+

CD27^+^	+	+

EBNA1	+	+

LMP1	+	+

EBNA2	+	+

### IL6 is expressed by LMP1^+ ^and LMP1^- ^cells

Figure 6D shows that after exposure of sorted memory or naïve B cells (as in Figure 5) to EBV, 61.6% of memory B cells and 19.2% of naïve B cells were LMP1-positive. Within memory cells, 30.7% (18.9/[18.9 + 42.7]) of LMP1^+ ^cells expressed IL6 while 25.8% (9.9/[9.9 + 28.5]) of LMP1^- ^cells expressed IL6. Although LMP1^+ ^cells contributed to two-thirds of the IL6-expressing population, LMP1^- ^cells contributed to the other third. It is unclear whether this latter population is infected with EBV. Nearly 20% of naïve B cells expressed LMP1; yet no IL6 expression was observed. Among memory B cells, IL6-producing cells were CD23^lo^, non-proliferating cells (Figure 5B) and LMP1^+ ^cells largely contributed towards IL6 expression (Figure 6D), suggesting that the majority of IL6-producing cells were non-proliferating, CD23^lo^, LMP1^+^, memory B cells.

## Discussion

This study focuses on early cellular events following encounter of B cells with EBV. EBV differentially infects B cells such that sub-populations of cells with distinct phenotypic and functional characteristics emerge. While expression of CD23, CD58, and IL6 have been examined individually either after infection with EBV [6,7,20,21] or after transfection of EBV latency gene LMP1 [10], to our knowledge, this is the first report of identification of sub-populations of B cells based on co-expression of these molecules, marking EBV-infected cells early for different outcomes. The expression pattern CD23^hi^CD58^+^IL6^- ^predicts the identity of infected cells destined for proliferation as early as three days after exposure to EBV. Another sub-population of CD23^+ ^B cells, also infected with EBV and expressing EBNA2 and LMP1, expresses IL6 but fails to proliferate.

Earlier investigations have shown that only cells co-expressing Epstein-Barr nuclear antigens and CD23 undergo immortalization [11]. These investigations did not differentiate between the different EB nuclear antigens. Our experiments demonstrate that expression of EBV latency genes, LMP1 and EBNA2, and CD23, are not sufficient for proliferation. While low levels of LMP1 transcripts relative to EBNA2 transcripts in CD23^lo^CD58^+ ^cells suggested lack of transition to high levels of expression of LMP1 as a potential reason for their inability to proliferate, LMP1 protein expression levels in individual cells did not substantiate this possibility. Greater than 80% of CD23^lo^CD58^+ ^cells expressed LMP1 at levels comparable to those observed in CD23^hi^CD58^+ ^cells. Furthermore, the *LMP1 *gene product appeared to be a latency protein since we did not observe expression of lytic gene *BZLF1 *in any of the sub-populations of cells (data not shown). Thus, B cell differentiation, abundance of expression of CD23, and other cellular determinants are among the likely causes of non-proliferation of CD23^lo^CD58^+ ^cells. Whether the cell surface molecular expression patterns that characterize the two sub-populations are causal to the distinct outcomes, have other functional significance, or simply mark the sub-populations is unclear.

We initially infected total peripheral blood B cells with high titers of EBV to include both naïve and non-naïve B cells that are thought to be targets of infection during primary infection with EBV and during the early stages of development of B cell-EBV lymphomas in immunocompromised hosts. To better understand whether the dichotomy in outcome was related to the differentiation stage of target B cells, we exposed purified naïve and memory B cells to EBV. Proliferating and non-proliferating EBV-infected cells did not exclusively derive from one or the other type of B cells. A related question, which has been the subject of earlier investigations, is whether memory B cells serve as direct targets for EBV-mediated proliferation and immortalization. Based on experiments that have relied on expression of viral latency genes for evidence of "immortalization", it has been extrapolated that EBV can immortalize both memory and naïve B cells [2,3,22]. However, using viral latency gene expression as evidence for immortalization may be fallacious as our data demonstrates that expression of viral latency genes and CD23 during the early stages of EBV infection does not necessarily correlate with proliferation and therefore potentially immortalization. We have found that while EBV infects both memory and naïve B cells (Figure 6D) *ex vivo*, using the marker pattern that we have identified, it appears that memory cells can serve as direct targets for EBV-driven proliferation (Figure 5). The inability of LMP1^+^-naïve B cells to proliferate in our study may be related to the absence of other types of cells or cytokines such as IL6 in an *ex vivo *setting.

EBNA2 RNA was the only latency gene product detected in CD23^lo^CD58^- ^cells. Since EBNA2 is not easily amenable to FACS staining, we were unable to determine if EBNA2 protein was expressed and if so, in what fraction of this sub-population, and at what levels. However, since EBNA2 is a major transactivator of LMP1 in the early stages of infection [23,24], low levels of expression of LMP1 protein in a very small fraction of CD23^lo^CD58^- ^cells may serve as an indirect indicator of deficient EBNA2 protein. Low levels of expression of CD23 within cells in this sub-population may also contribute to lack of cell proliferation. Certainly lack of expression of CD23 in EBNA^+ ^cells is known to prevent immortalization of B cells [6,7].

IL6 is predominantly expressed by CD23^lo^CD58^+^LMP1^+ ^cells. Expression of IL6 by non-proliferating cells is consistent with the observation that only rare EBV-immortalized tonsillar blasts expressed IL6 during primary infection [25]. IL6 is a growth factor for LCL in culture [20] and in SCID mice [26]. A strong positive correlation exists between development of post-transplant EBV-lymphomas in humans and elevated levels of serum IL6 [27]. It is tempting to speculate that IL6-producing cells aid the proliferating sub-population, perhaps during the early stages of EBV-infection via paracrine mechanisms. While the results of the experiment in Figure 4 argue in favor of this hypothesis, post-sort mixing experiments will be necessary to determine the effects of the different sub-populations and IL6 on the viability and proliferation of CD23^hi^CD58^+ ^cells.

Transfection of *LMP1 *gene was found to increase expression of CD58 [9], an adhesion molecule, suggesting that LMP1 drives expression of CD58 following EBV infection. We observed a discernible increase in the fraction of CD23^+^CD58^+ ^cells as early as 18 h after exposure to EBV (Table 1); yet, LMP1 expression has not been detected earlier than 48 h after exposure to EBV [28]. This raises the question of an LMP1-independent mode of expression for CD58. In support of an LMP1-independent mode of expression of CD58, we found that greater than 50% of CD23^- ^cells expressed CD58 around day 3 (Table 1) while fewer than10% of CD23^- ^cells expressed LMP1 on day 3 (data not shown).

In the absence of EBV infection, CD58 was expressed almost exclusively on CD23^- ^cells (Table 1, time 0), while after exposure to EBV, expression of CD58 was up-regulated greatly on CD23^+ ^cells. Since only a fraction of CD23^+^CD58^+ ^cells proliferated, it is unlikely that CD58 plays a direct role in proliferation. CD58 may exert its effects in a broader and more global capacity to indirectly affect the proliferating population. Indeed this rapid up-regulation of CD58 may be part of the immune response alerting the host to infection with EBV. Interaction of CD58 with its ligand CD2 on T cells may elicit EBV-directed T cell responses. Interactions between B and T cells were noted following gene transfer of LMP1 [9].

Although using B cells from healthy EBV-seronegative individuals may have yielded two potential advantages, we used cells from healthy EBV-seropositive subjects. First, depletion of T cells might not have been necessary and second, "contaminating" naturally infected B cells, although very few in EBV-seropositive subjects, would have been absent in cells derived from EBV-seronegative individuals. However, since most EBV-seronegative individuals are children and adolescents, it is difficult to obtain large amounts of blood with each draw. As large volumes of blood were necessary for our experiments, the majority of experiments were performed with B cells from healthy EBV-seropositive individuals. To ensure that naturally infected B cells did not give rise to proliferating cells, we tested B cells from all five EBV-seropositive subjects for outgrowth in culture in the absence of T cells and exogenous EBV. None of the cells showed outgrowth as shown for one subject in Figure 3C. Thus, although 1-50 out of 10^6 ^peripheral B cells in EBV-seropositive subjects are naturally infected with EBV [29], these cells are unable to grow out in culture even in the absence of T cells. Experiments with cells from two EBV-seronegative subjects demonstrated emergence of similar sub-populations of B cells after exposure to EBV (Figure 2B).

Consistent with the work of others [30], between 5% (Figure 1A) and 15% (data not shown) of peripheral B cells are CD23^lo ^while the rest are CD23^- ^prior to exposure to EBV. Mature B cells express low levels of CD23 [31]. Whether the initial drop in the fraction of CD23^lo ^cells following exposure to EBV (Figure 1A) is due to down-regulation of cell surface expression of CD23 or death of CD23-expressing cells is unclear. Since CD27^+ ^memory B cells tend to be CD23^- ^[32] and give rise to CD23^hi ^cells (Figure 5B), it is likely that CD23^hi ^cells derive from CD23^- ^cells. This is consistent with the work of Azim et al. which suggests that CD23^- ^cells could serve as targets for EBV-mediated immortalization [33].

## Conclusions

Our findings delineate some of the earliest events after exposure to EBV and identify a sub-population of EBV-infected B cells predicted to undergo proliferation as early as three days after exposure to EBV. Additional factors, especially those of cellular origin, are likely to be important in determining whether an EBV-infected B cell expressing viral latency genes undergoes proliferation. The ability to selectively examine cells at earliest times as they commit to proliferation will be key to understanding how EBV converts a B cell into a cell with oncogenic potential. Comparative analyses between B cells destined for or refractory to EBV-driven proliferation will yield new avenues of investigation into EBV pathogenesis and potentially EBV therapeutics.

## Methods

### Isolation of B cells

Peripheral Blood Mononuclear Cells (PBMC) were obtained from healthy adults (five males and two females) as described [34]. The use of human subjects was approved by the Human Investigation Committee at Yale University. Informed written consent was obtained from volunteers. EBV-seroreactivity was determined by presence of antibodies to EBNA1 and viral capsid antigen using Western Blot. Experiments were performed with cells from five EBV-seropositive and two EBV-seronegative individuals. Experiments were repeated with cells from up to six subjects. B cells were isolated using negative selection by immunomagnetic-depletion of CD3^+ ^cells (Invitrogen) with the exception of experiments in Figure 5 and Figure 6D in which B cells were positively selected and sorted by FACS. Negative selection of B cells was performed to avoid inadvertent activation of B cells during isolation. At least 95% of CD19^+ ^B cells expressed CD21, the receptor for EBV (data not shown). Monocytes were depleted by adherence to plastic ([35]; data not shown).

### EBV preparation and infection of B cells

EBV was isolated from the supernatant of B95-8 cells as described [36]. Infectivity of virus preparations was assessed by infection in triplicate of EBV-negative BJAB cells with serial dilutions of virus. After 48 h of culture, cells were examined for expression of EBNA by indirect immunofluorescence as described [37] and virus titer was calculated. Infections were performed using titered EBV at multiplicity of infection of 50-100 to maximize the number of infected B cells. After incubation of cells with virus for two hours at 37°C, cells were washed twice and placed in culture in the presence of 5% CO_2 _at 2 × 10^6 ^ml^-1 ^in RPMI 1640 containing 10% FBS.

### CFSE labeling of cells

Carboxyfluorescein diacetate, succinimidyl ester (CFSE; Invitrogen) labeling of un-infected B cells was performed as described [38]. We had experimentally determined that 2 μM CFSE allowed detection of proliferation for up to four generations with minimal toxicity to cells (data not shown).

### Flow cytometric examination and sorting of cells

Cells were surface-stained with saturating concentrations of antibodies including anti-CD23-PE (BD Bioscience), anti-CD23-FITC (Dako), anti-CD23-biotin (BD Bioscience), anti-CD58-FITC (ABD Serotec), anti-CD58-PE-Cy5 (Biolegend), anti-CD58-biotin (Gene Tex, Inc.), anti-CD86-APC (BD Bioscience), anti-CD57-FITC (BD Bioscience), anti-MHC Class II- FITC (BD Bioscience), anti-PD1-APC (eBioscience), and anti-CD27-biotin (Biolegend). Murine Ig at 1 mg ml^-1 ^was included to inhibit nonspecific binding. Isotype-matched control antibodies included murine IgG1-PE, IgG1-FITC, IgM-FITC, IgG2a-FITC, IgG1-APC, IgG1-PE-Cy5, and IgG1-biotin. Biotinylated antibodies were detected using Avidin-PE-Cy7 (BD Bioscience) or Avidin-FITC (Zymed). For intracellular staining, cells were fixed and permeabilized with Cytofix/Cytoperm (BD Pharmingen) or 90% cold methanol (for LMP1 staining). Antibodies included anti-IL6-FITC (eBioscience), anti-IL6-APC (Biolegend), anti-IL10 Pacific blue (eBioscience), and anti-LMP1 (CS1-4, Dako) followed by anti-mouse IgG1-PE or FITC. Isotype control antibodies included murine IgG2b-FITC, rat IgG1κ-APC, rat IgG1-Pacific blue, and murine IgG1. Data was acquired on LSR II (BD Bioscience) or FACS Calibur and analyzed using WinMDI. Gates were set on live lymphocytes based on forward- and side-scatter profiles. Quadrants or gates were drawn after comparing cells stained with an antibody of interest to cells stained with a matched isotype control antibody. For analysis of cell proliferation, gates were manually drawn on live cells for each CFSE peak as described [39,40]. Unlabeled but EBV-exposed cells and labeled but un-infected cells were used as controls. Sorting was performed on FACS Vantage or Aria Cell Sorter.

### PCR and qRT-PCR

Primer sequences targeting the BamW fragment of EBV were: Forward: 5'GACTCCGCCATCCAAGCCTAG3'; Reverse: 5'TGGACGAGGACCCTTCTACGG3'.

Relative transcript levels of EBV latency genes were determined by real-time reverse transcription-PCR (qRT-PCR) of cDNA from sorted cells using gene-specific primers. Primer sequences were: EBNA1: Forward: 5'AGGGGAAGCCGATTATTTTG3';

Reverse: 5'CTCCTTGACCACGATGCTTT3'; LMP1: Forward: 5'TGAGTGACTGGACTGGAGGA3'; Reverse: 5'GGCTCCAAGTGGACAGAGAA3'; EBNA2: Forward: 5'CGGTCCCCGACTGTATTTTA3'; Reverse: 5'GGCTCTGGCCTTGAGTCTTA3'. Relative expression levels were calculated using standard curves generated from serial dilutions of EBV^+ ^Akata Burkitt Lymphoma cell total RNA and normalized to 18 S rRNA.

## Competing interests

The authors declare that they have no competing interests.

## Authors' contributions

CM performed all experiments except qRT-PCR. JY performed qRT-PCR experiments. SBM designed the study, performed experiments, analyzed data, interpreted results, and wrote the manuscript. All authors read and approved the final manuscript.
